# The Dopamine Metabolite 3-Methoxytyramine Is a Neuromodulator

**DOI:** 10.1371/journal.pone.0013452

**Published:** 2010-10-18

**Authors:** Tatyana D. Sotnikova, Jean-Martin Beaulieu, Stefano Espinoza, Bernard Masri, Xiaodong Zhang, Ali Salahpour, Larry S. Barak, Marc G. Caron, Raul R. Gainetdinov

**Affiliations:** 1 Department of Neuroscience and Brain Technologies, Italian Institute of Technology, Genova, Italy; 2 Department of Cell Biology, Duke University Medical Center, Durham, North Carolina, United States of America; 3 Department of Psychiatry and Neuroscience, CRULRG/Université Laval, Québec, Canada; 4 INSERM U 858 - I2MR, Toulouse, France; 5 Neuroscience and Behavioral Disorders Program, Duke-National University of Singapore Graduate Medical School, Singapore, Singapore; 6 Department of Pharmacology and Toxicology, University of Toronto, Toronto, Canada; University of Minnesota, United States of America

## Abstract

Dopamine (3-hydroxytyramine) is a well-known catecholamine neurotransmitter involved in multiple physiological functions including movement control. Here we report that the major extracellular metabolite of dopamine, 3-methoxytyramine (3-MT), can induce behavioral effects in a dopamine-independent manner and these effects are partially mediated by the trace amine associated receptor 1 (TAAR1). Unbiased *in vivo* screening of putative trace amine receptor ligands for potential effects on the movement control revealed that 3-MT infused in the brain is able to induce a complex set of abnormal involuntary movements in mice acutely depleted of dopamine. In normal mice, the central administration of 3-MT caused a temporary mild hyperactivity with a concomitant set of abnormal movements. Furthermore, 3-MT induced significant ERK and CREB phosphorylation in the mouse striatum, signaling events generally related to PKA-mediated cAMP accumulation. In mice lacking TAAR1, both behavioral and signaling effects of 3-MT were partially attenuated, consistent with the ability of 3-MT to activate TAAR1 receptors and cause cAMP accumulation as well as ERK and CREB phosphorylation in cellular assays. Thus, 3-MT is not just an inactive metabolite of DA, but a novel neuromodulator that in certain situations may be involved in movement control. Further characterization of the physiological functions mediated by 3-MT may advance understanding of the pathophysiology and pharmacology of brain disorders involving abnormal dopaminergic transmission, such as Parkinson's disease, dyskinesia and schizophrenia.

## Introduction

The phenylethylamine derivative dopamine (DA) plays an important role in the brain as a neurotransmitter that mediates many critical functions including motor control [1], [2], [3]. During DA synthesis, L-DOPA is produced from the amino acid tyrosine by tyrosine hydroxylase (TH) and further decarboxylated by L-aromatic acid decarboxylase (L-AADC) to yield DA [1], [2]. Synthesized DA is accumulated in synaptic vesicles [4], [5], thus becoming available for release into the extracellular space. After release and activation of its receptors, DA undergoes dilution by diffusion, but also becomes subject to metabolic degradation by catechol-o-methyl transferase (COMT) [1], [6], [7]. This process yields the major extracellular metabolite, 3-methoxytyramine (3-MT), that is generally considered to be a biologically inactive compound. At the same time, a large portion of released DA is re-captured into dopaminergic terminals by the plasma membrane dopamine transporter (DAT) [8], [9], thus providing a large intracellullar storage pool of recycled DA available for subsequent re-release [10], [11].

By using mice lacking the dopamine transporter (DAT-KO mice) [9], [11], we have developed a model of acute dopamine deficiency, DDD mice (dopamine-deficient DAT-KO mice) [12], [13]. In the absence of DAT-mediated recycling mechanisms in DAT-KO mice, neuronal DA levels become entirely dependent on its de novo synthesis [10], [11]. Pharmacological inhibition of DA synthesis in these mice by the irreversible inhibitor of TH α-methyl-p-tyrosine (αMT) induces prolonged depletion of dopamine in the major dopaminergic regions of the brain such as striatum. This acute DA deficiency results in the development of a pronounced behavioral phenotype manifested as severe akinesia and rigidity. As expected, the movement in DDD mice can be restored by administration of the DA precursor L-DOPA or by treatment with non-selective DA agonists [12], [13]. We took advantage of having this simple and reversible model of severe DA deficiency to search for alternative treatments that can affect movement control in the absence of DA [12], [14]. Interestingly, several amphetamine derivatives counteracted behavioral manifestations of DA deficiency in DDD mice in a DA-independent manner. This led us to suggest that DA and DAT-independent targets of amphetamines may be responsible for these effects [12]. Among the several known targets of amphetamine derivatives, is the newly identified, G protein-coupled trace amine associated receptor 1 (TAAR1, also known as trace amine receptor 1, TA1) [15], [16], [17], [18], [19]. Intriguingly, TAAR1 can be activated *in vitro* not only by amphetamines but also by many other phenylethylamine derivatives including trace amines themselves and monoamine metabolites, including 3-MT [16], [20], [21], [22]. While biogenic trace amines such as tyramine, tryptamine, β-phenylethylamine, octopamine and synephrine are found in various tissues in many species in the kingdom *Animalia*, only tyramine and octopamine have been recognized as neurotransmitters in invertebrates. Both of these are critically involved in regulation of various physiological functions such as circadian rhythms, emotional behaviors, cardiovascular regulation, learning, memory and movement [18], [23], [24]. To explore if trace amines or other endogenous compounds active on TAAR1 could be involved in movement control in mammals, we performed an unbiased screen of several compounds that activate TAAR1 for their potential effects on locomotor activity in akinetic DDD mice [12], [13]. Unexpectedly, we observed potent behavioral and biochemical effects of the dopamine metabolite 3-MT that were partially dependent on TAAR1. These observations indicate that 3-MT is not just an inactive metabolite of DA but a neuromodulator that may play a role of its own in motor control.

## Methods

### Animals

DAT-KO and TAAR1 knockout (TAAR1-KO) mice of mixed C57BL/6J x 129Sv/J backgrounds were generated as described [9], [12]. All studies were conducted with approved protocols from the Duke University Institutional Animal Care and Use Committee and were in accordance with the NIH guidelines for the Care and Use of Laboratory Animals. 3–6 month old wild type (WT) and knockout (KO) mice of both sexes and male C57BL/6J mice were used in this study.

### Drugs

Compounds or saline (0.9% NaCl) were administered intraperitoneally (i.p.) or subcutaneously (s.c.) in a volume of 10 ml/kg or intracerebroventricularly (i.c.v.) in a volume of 4 µl. For i.c.v. administration compounds were dissolved in artificial cerebrospinal fluid and infused into the right lateral ventricle at a rate of 1 µl/min as described previously [25]. Corresponding vehicle solutions were infused to respective control animals. All the compounds used were from Sigma (St. Louis, MO).

### Behavioral methods

Locomotor activity of DAT-KO and WT mice was measured in an Omnitech CCDigiscan (Accuscan Instruments, Inc. Columbus, Ohio USA) monitor under conditions of bright illumination [12], [26]. Activity parameters were continuously monitored and tallied at 5 min intervals. To evaluate the effects of compounds in DDD mice, DAT-KO mice were placed into activity monitor chambers for 30 min and then treated systemically with αMT (250 mg/kg, i.p.). 1 h after αMT administration a compound or combination of drugs was injected systemically or i.c.v. and various parameters of locomotor activity were monitored for up to 3 h. In cumulative dosing experiments, animals were treated with increasing doses of compounds with a one hour interval. To assess effects of 3-MT in normal and TAAR1-KO mice, the animals were placed in the locomotor activity chamber and 30 min later various doses of 3-MT were administered i.c.v.. To perform i.c.v. administration in this paradigm, habituated mice were removed from the experimental chamber, briefly restrained, i.c.v. injection cannula was placed into the previously implanted (one week before) guide cannula and infusion of 3-MT or vehicle (artifical CSF) was performed for 4 minutes when animal was freely moving in a home cage. After infusion, animals were put back into experimental chamber and behavior was monitored for 90 min after administration.

### *In vitro* transfection of human TAAR1 and cAMP Assay

All cell culture reagents and buffers were from Gibco and Sigma. Human embryonic kidney 293 (HEK-293) cells were maintained in Minimum Essential Medium Eagle (MEM) medium supplemented with 10% (vol/vol) of FBS, 2 mM glutamine and 0.05 mg/ml of Gentamicin at 37°C in a humidified atmosphere at 95% air and 5% CO2. Transient transfections were performed 24 h after cell seeding using calcium phosphate protocol. A modified version of human TAAR1(hTAAR1) in which the first nine amino acids of the β2-adrenergic receptor were added at N-terminus of hTAAR1 to enable plasma membrane expression of the receptor was used as previously described [21]. 5 µg of hTAAR1 or of an empty vector for each ml of transfection solution were used. To investigate effects of tyramine and 3-MT at hTAAR1 we measured the cAMP response using the standard cAMP column assay [21], [27].

### Antibodies and Western Blot Analyses

The antiphospho-ERK1/2 (Thr-202/Tyr-204), anti-ERK, anti-phospho-CREB (Thr-34) and anti-CREB antibodies were purchased from Cell Signaling Technology (Beverly, MA). Western blot analyses of brain samples were performed as described in Beaulieu *et al.* [28]. Briefly, mice were euthanized by decapitation, after which the heads of the animals were immediately cooled by immersion in liquid nitrogen for 6 s. The right hemisphere striatum was rapidly dissected (within 60 s) on an ice-cold surface and frozen in liquid nitrogen before protein extraction. Tissue samples were homogenized in boiling 1% SDS solution and boiled for 10 min. Protein concentrations were measured using a DC-protein assay (Bio-Rad, Hercules, CA). Protein extracts (25 or 50 µg) were separated on 10% SDS/PAGE and transferred to nitrocellulose membranes. Blots were incubated with primary antibodies overnight at 4°C. Immune complexes were detected using appropriate peroxidase-conjugated secondary antibodies (Jackson Immuno-Research, West Grove, PA) and a chemiluminescent reagent (SuperSignal West-Pico; Pierce Biotechnology, Rockford, IL). Densitometric analysis was performed within the linear range using IMAGEQUANT V1.1 (GE Healthcare Life Sciences, Piscataway, NJ). For quantitative analysis, total proteins were used as loading controls for phosphoprotein signals. In all these experiments, results were normalized to respective controls and presented as means ±SEM.

To analyze effect of 3-MT on TAAR1-mediated intracellular signaling events in HEK-293 cells, hTAAR1 was expressed as described [21]. After 24 of transfection, cells were lysed with RIPA buffer supplemented with protease (Roche Diagnostic) and phosphatase (Thermo Scientific) inhibitors. After 10 minutes of incubation on ice, lysates were centrifuged for 10 minutes at 13000 rpm and supernatants were collected for protein concentration assay (BCA protein assay kit, Thermo Scientific). 25 µg of protein extract were separated on 10% SDS/PAGE and transferred on nitrocellulose membrane. All primary antibodies were incubated overnight at 4°C. Appropriate peroxidase-conjugate secondary antibodies (Pierce) and chemiluminescent reagents (ECL detection reagent, Amersham) were used. Total protein levels were used as loading controls for phosphoprotein signals. Results were normalized to respective controls.

### Data analysis

The data are presented as means ± SEM and analyzed using a two-tailed Student's t-test, one-way ANOVA followed by Dunnet's multiple comparison test, two-way ANOVA followed by Tukey's HSD test or a two-tailed Mann-Whitney U-test where appropriate.

## Results

### Unbiased screen of trace amines and monoamine metabolites for potential effects on movement in DDD mice

**Figure 1:**
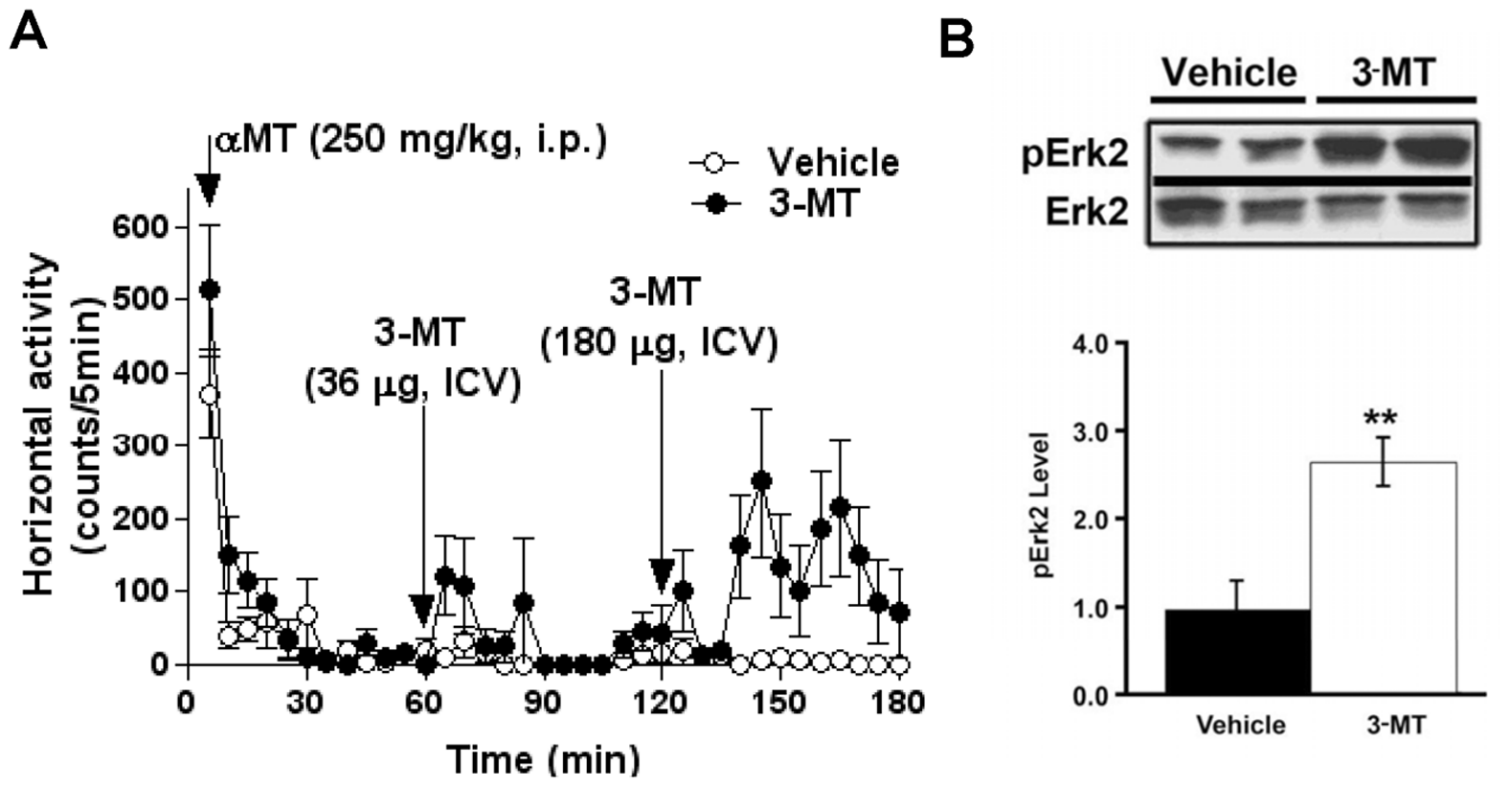
3-MT induces behavioral activation and intracellular signaling in the striatum of DA deficient mice. (A) Identification of motor actions of 3-MT in DDD mice. DAT-KO mice were treated with αMT (250 mg/kg, i.p.) and 1 h after αMT were challenged repeatedly with increasing concentrations of a drug (interval between treatments is 1 h). 3-MT induced abnormal activation in DDD mice after i.c.v. infusion of both 36 and 180 µg of 3-MT (visual observations), as revealed by the significant effect of 3-MT in measures of horizontal activity at dose 180 µg (cumulative horizontal activity counts for 1 h following infusion of 180 µg 3-MT is 1711.4±580.1 vs. 26.8±11.8 in vehicle-treated group; p<0.05, two-tailed Mann-Whitney U test, n = 6 per group). (B) 3-MT administered at dose of 36 µg, i.c.v. (30 min after infusion) caused significant increase in Erk2 phosphorylation in the striatal tissue of DDD mice (n = 10 per group; ** - p<0.01; Student's t-test).

The ability of α-methyl-*p*-tyrosine (αMT), a potent irreversible inhibitor of TH, to selectively deplete brain DA in mice lacking the DAT provided a simple *in vivo* model of DA deficiency to use as a test system to analyze DA-independent actions of various compounds [12]. It should be noted, that while the global nature of DA deficiency may limit the use of this model in studies aimed at deciphering particularities of brain circuitry at normal conditions, it provides certain advantages in studies aimed at uncovering DA-independent mechanisms involved in motor control. Thus, we elected to employ this model to perform an unbiased screen of putative TAAR1 ligands and monoamine metabolites for potential actions on movement control. Briefly, DAT-KO mice were treated with αMT and 1 hour later, when maximal depletion of DA was achieved the tested compounds were administered. In an initial screening, no significant effect on motor control was observed when these compounds were administered systemically (intraperitoneal injection; i.p.) [12]. Since many of these compounds have limited capacity to pass the blood-brain barrier (BBB), we administered them intracerebroventricularly (i.c.v.) using a paradigm of successively increasing the concentration given to a single animal (Table 1). The trace amines p-tyramine, m-tyramine, octopamine, tryptamine, β-phenylethylamine (β-PEA) and monoamine metabolites 4-methoxytyramine (4-MT), metanephrine, normetanephrine, 3,4-dihydroxyphenylacetic acid (DOPAC) and homovanillic acid (HVA) caused no significant effect in akinetic DDD mice (Table 1, data not shown). Unexpectedly, the extracellular DA metabolite 3-methoxytyramine (3-MT) induced significant behavioral activation in DDD mice (Figure 1A). This activity however, was mostly presented as a set of disorganized abnormal movements that included tremor, head bobbing, straub tail, grooming and abnormal orofacial movements rather than normal forward activity. To verify that this endogenous compound can affect a receptor-mediated cellular signaling mechanism we collected striatal tissue of DDD mice treated with 36 µg of 3-MT (30 min after 3-MT administration) and performed analysis of ERK activity by Western blot. We elected to analyze ERK signaling as it represents one of the most common signaling mechanisms involved in multiple striatal functions, including movement control [29], [30], [31]. As presented in Figure 1B, 3-MT caused a significant increase in the level of phosphorylated Erk2, thus indicating that certain receptor-mediated processes in striatal cells are affected by this treatment.

**Table 1:** Trace amines and monoamine metabolites tested in DDD mice.

Trace amines and monoamine metabolites	Doses	Number of mice	Motor effects
p-Tyramine	36 and 360 µg, i.c.v.	4	No
m-Tyramine	36, 72 and 180 µg, i.c.v.	4	No
Octopamine	36 and 360 µg, i.c.v.	4	No
Tryptamine	36, 72 and 180 µg, i.c.v.	4	No
β-Phenylethylamine	100 µg, i.c.v.	4	No
	200, 400 µg, i.c.v.	4	No
4-Methoxytyramine (4-MT)	18, 72 and 180 µg, i.c.v.	4	No
Metanephrine	18, 72 and 180 µg, i.c.v.	4	No
Normetanephrine	18, 72 and 360 µg, i.c.v.	4	No
DOPAC	18, 72 and 360 µg, i.c.v.	4	No
HVA	27 and 68 µg, i.c.v.	4	No
3-methoxytyramine (3-MT)	36 and 180 µg, i.c.v.	6	Yes

Compounds were administered i.c.v. 1 h after αMT treatment (250 mg/kg, i.p.). In cumulative dosing experiments, animals were treated with drugs with interval 1 h.

Furthermore, to directly explore if these motor effects could be mediated via the postulated activation of DA receptors by 3-MT at high concentrations [48], [49], we pre-treated an additional group of DDD mice with a combination of D2 DA receptor antagonist raclopride (2 mg/kg, i.p.) and D1 DA receptor antagonist SCH-23390 (0.1 mg/kg, i.p.) 30 minutes before 3-MT (90 µg) infusion. Infusion of 3-MT at this behaviorally active concentration (Figure 1A) to saline pre-treated DDD mice caused abnormal movements as evidenced by significantly increased horizontal activity counts in 1 hour period (1097±459 in saline pre-treated 3-MT infused group vs. 50±27 in saline pre-treated vehicle infused controls, * p<0.05; Student's t-test n = 4-6 per group). Since DDD mice demonstrate spontaneous severe akinesia and rigidity [12], pre-treatment with DA antagonists did not induce additional locomotor effects in vehicle infused controls. Importantly, pre-treatment with DA antagonists did not affect the ability of 3-MT to induce abnormal movements (horizontal activity counts/1 hour: 1259±661 in DA antagonists pre-treated 3-MT infused group vs. 24±2 in DA antagonists pre-treated vehicle infused controls, * p<0.05; Student's t-test, n = 4–6 per group). By comparison, the same pre-treatment protocol involving combination of raclopride and SCH-23390 at relatively high doses completely abolished locomotor-restoring effect of L-DOPA/carbidopa (50/50 mg/kg, i.p.) in DDD mice [12]. Thus, DA receptors are unlikely to be involved in the observed locomotor effects of 3-MT.

To verify if 3-MT is able to cause similar effects in wild type mice with an intact DA system we tested several doses of 3-MT in C57Bl6 mice. While no effect was observed when 3-MT was infused at doses below 9 µg (data not shown), at 9 µg and higher doses 3-MT dose-dependently caused transient behavioral activation with a complex set of behaviors. In particular, transient hyperactivity and stereotypy, sniffing, grooming, rearing and mild abnormal involuntary movements (AIMs) at the level of limbs was observed after infusion of 9 µg of 3-MT. Similar behaviors were also observed after 18 µg of 3-MT with the additional appearance of tremor as well as oral and whole body AIMs. Further progression of these behaviors to a complex phenotype involving head bobbing, backward walking, prominent orofacial and whole body AIMs as well as minor seizure activity was found with 36 µg of 3-MT. Infusion of higher concentrations of 3-MT caused pronounced seizures in normal mice (data not shown). Examples of behavioral effects of 3-MT (36 µg, 40 min after administration) in normal mice of mixed 129SvJ/C57BL6 background (wild type littermate controls for TAAR1-KO mice) are presented in the Supplemental Videos S1 and S2. Given the extreme complexity and quickly changing nature of this behavioral phenotype in mice we elected to perform unbiased computerized assessment of abnormal movement behaviors in locomotor activity chambers, rather than just simply applying ethological scoring approaches developed for other manifestations of abnormal behavioral activation, such as stereotypies and dyskineisas. Dynamics of behavioral effects of 3-MT in C57BL6 mice at doses 9–36 µg as detected in a computerized locomotor activity monitor by changes in total distance traveled, vertical activity and stereotypy time are presented in the Supplemental Figure S1. Nevertheless, future development of specific ethological scoring system will be necessary to perform careful characterization of these abnormal behaviors induced by 3-MT in more details.

In addition, to estimate the brain extracellular concentration of 3-MT that could be achieved after i.c.v. administration of 3-MT in C57BL6 mice, we performed *in vivo* microdialysis measurements of 3-MT in the dialysates collected from striatum of freely moving mice following 3-MT infusion into the lateral ventricle. As presented in the Supplemental Figure S2, i.c.v. infusion of 3-MT (9 µg) caused potent elevation of 3-MT concentrations in striatal dialysates with maximal levels approaching 100 nM.

### 3-MT activates TAAR1-mediated signaling *in vitr*o

**Figure 2:**
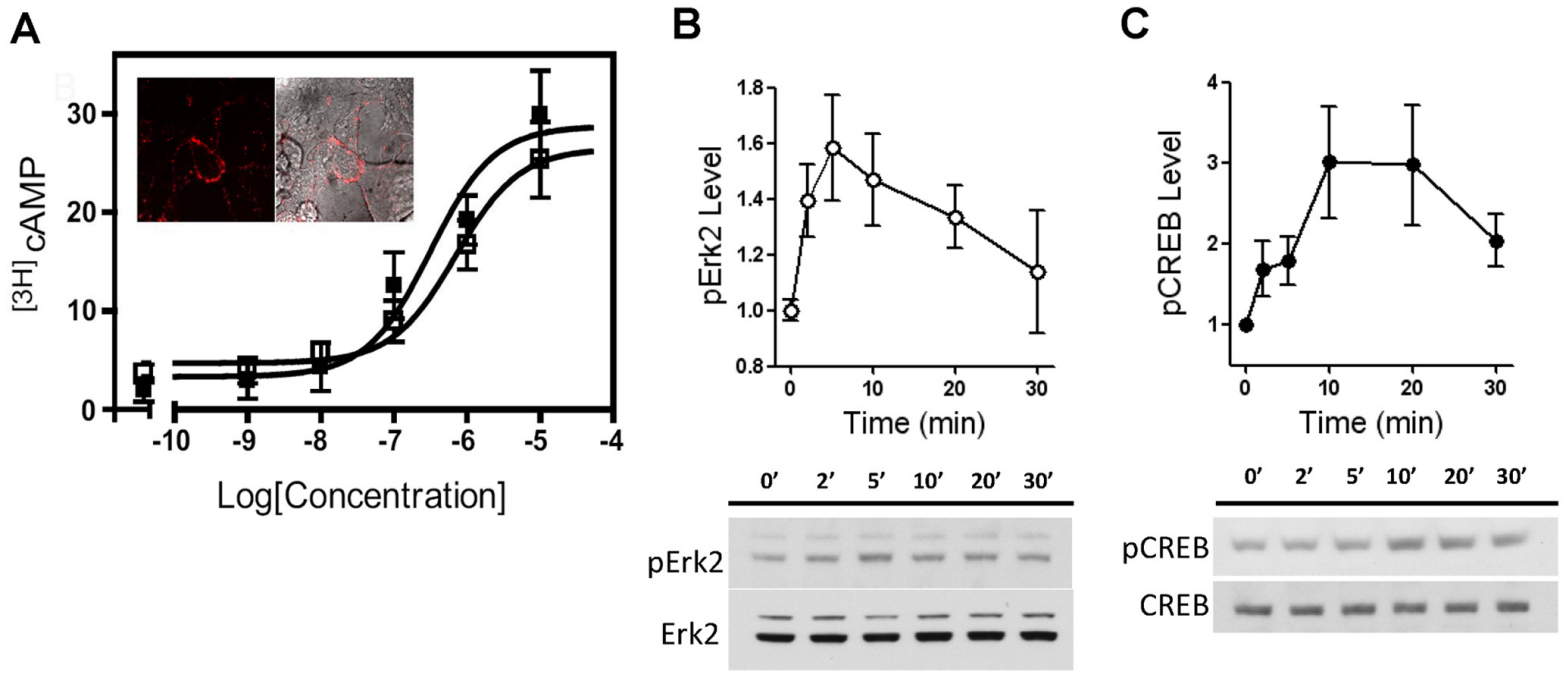
3-MT induces activation of human TAAR1 in cAMP assay and causes CREB and Erk2 phosphorylation in HEK-293 cells. (A) cAMP response to tyramine and 3-MT in cells expressing hTAAR1 receptor. Dowex and Alumina column chromatography was used to measure [3H]-cAMP accumulation in HEK-293 cells transfected with the hTAAR1 receptor and treated with the concentrations of compounds shown in the Figure for 15 minutes at room temperature. Results are the mean ± SEM of two (tyramine) or three (3-MT) independent experiments performed in duplicate. EC50 for tyramine was estimated as 320±100 nM and for 3-MT as 700±180 nM. No effects of tyramine and 3-MT were observed in corresponding Mock cells expressing endogenous receptors only (data not shown). The inserted images obtained with a Zeiss LSM510 confocal microscope show the fluorescence from the immunostaining of HA epitope tagged hTAAR1 receptors expressed at the plasma membrane compartment of non permeabilized HEK-293 cells (left image), and an overlay of the fluorescence on a phase image of the same cells (right image) [21]. (B) and (C) Time-course of effect of 3-MT (10 µM) on Erk2 (B) and CREB (C) phosphorylation in HEK-293 cells expressing hTAAR1. hTAAR1 was expressed in cells as described previously [21] and treated with vehicle or 3-MT (10 µM). The cells were lysed and then analyzed by Western blot for Erk2 and CREB phosphorylation. 3-MT produced time dependent phosphorylation of Erk2 and CREB in cells expressing hTAAR1 while no effects were observed in vehicle-treated controls. A significant effect in comparison to untreated cells (time point 0) was observed at 2, 5, 10 and 20 min time points for ERK2 phosphorylation and at 10 and 20 min periods for CREB phosphorylation (n = 4 independent experiments per group, p<0.05, one-way ANOVA followed by Dunnet's multiple comparison test). No effect of 3-MT was observed in corresponding Mock cells without hTAAR1 expression (data not shown).

Since the discovery of the family of trace amine associated receptors (TAARs) [15], [16] most interest has been focused on the TAAR1. This Gs-coupled receptor has tantalizing pharmacology and can be activated, at least *in vitro*, by trace amines, amphetamines and several monoamine metabolites [18], [19]. It has been reported that 3-MT is an agonist of mouse, rat as well as rat-human and mouse-human chimeric versions of this receptor in cellular systems that express TAAR1 mostly intracellularly [15], [16], [17], [20], [22]. To confirm that 3-MT is able to activate human TAAR1, we employed a recently developed approach to express hTAAR1 at the plasma membranes of HEK cells [21]. Assessment of cAMP accumulation using a bioluminescence resonance energy transfer (BRET) assay [21] and a classical column cAMP assay (Figure 2) confirms activity of 3MT at TAAR1 with a potency comparable to tyramine (EC50 for 3-MT is 700±180 nM and for tyramine is 320±100 nM) (Figure 2A). Furthermore, we performed an analysis of intracellular signaling mechanisms that could mediate the actions of 3-MT on hTAAR1 expressed in HEK cells. 3-MT caused a rapid and prolonged phosphorylation of Erk2 and CREB only in cells expressing hTAAR1 (Figure 2B,C) thus indicating that a Gs-dependent signaling cascade involving accumulation of cAMP and activation of Erk2 and CREB is likely involved in the action of TAAR1.

### Effects of 3-MT are reduced in TAAR1-KO mice

**Figure 3:**
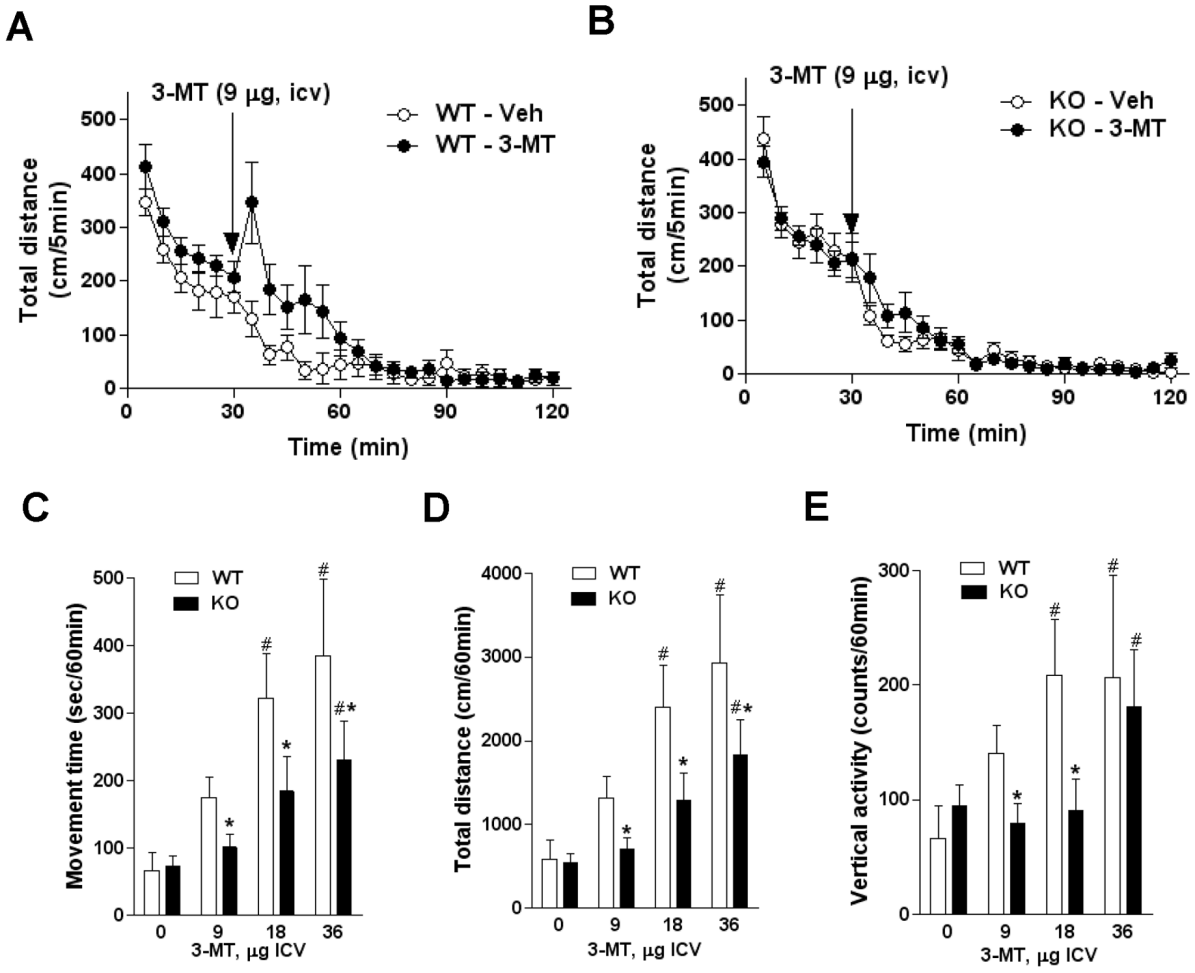
Behavioral effects of 3-MT are reduced in TAAR1-KO mice. Administration of 3-MT (9 µg, i.c.v.) to WT mice (A) but not TAAR1-KO mice (B) induced abnormal behavioral activation as reflected by total distance traveled. Analysis of total distance traveled for 60 min after 3-MT administration revealed significant effect of 3-MT versus vehicle treatment (p<0.05; Student's t-test) in WT but not TAAR1-KO mice (Figure 3D). (C, D and E) Dose-dependence of 3-MT-induced complex set of abnormal movements (please see description in the text and Supplemental Figure 1S) as detected in computerized locomotor activity monitor as changes in movement time (C), total distance traveled (D) and vertical activity (E). Data are presented as cumulative counts for 60 min after 3-MT administration. Two-way ANOVA analysis revealed significant main effects of dose (p = 0.0001) and genotype (p<0.0001), but no significant dose by genotype interaction (p = 0.4) in measures of movement time (C), significant main effects of dose (p = 0.0002), genotype (p<0.0001) and dose by genotype interaction (p = 0.0198) in measures of total distance (D) and significant main effects of dose (p = 0.026) and genotype (p<0.0001) but no significant dose by genotype interaction (p = 0.321) in measures of vertical activity (E). Pair-wise comparisons conducted with post-hoc Tukey's HSD test revealed significant differences between genotypes (* - p<0.05 effect of 3-MT in WTs vs. KOs) and dose (# - p<0.05 effect of 3-MT vs. respective vehicle-treated controls). Please note, that after 36 µg a similar pattern of abnormal movements was observed in both genotypes (visual observations).

**Figure 4:**
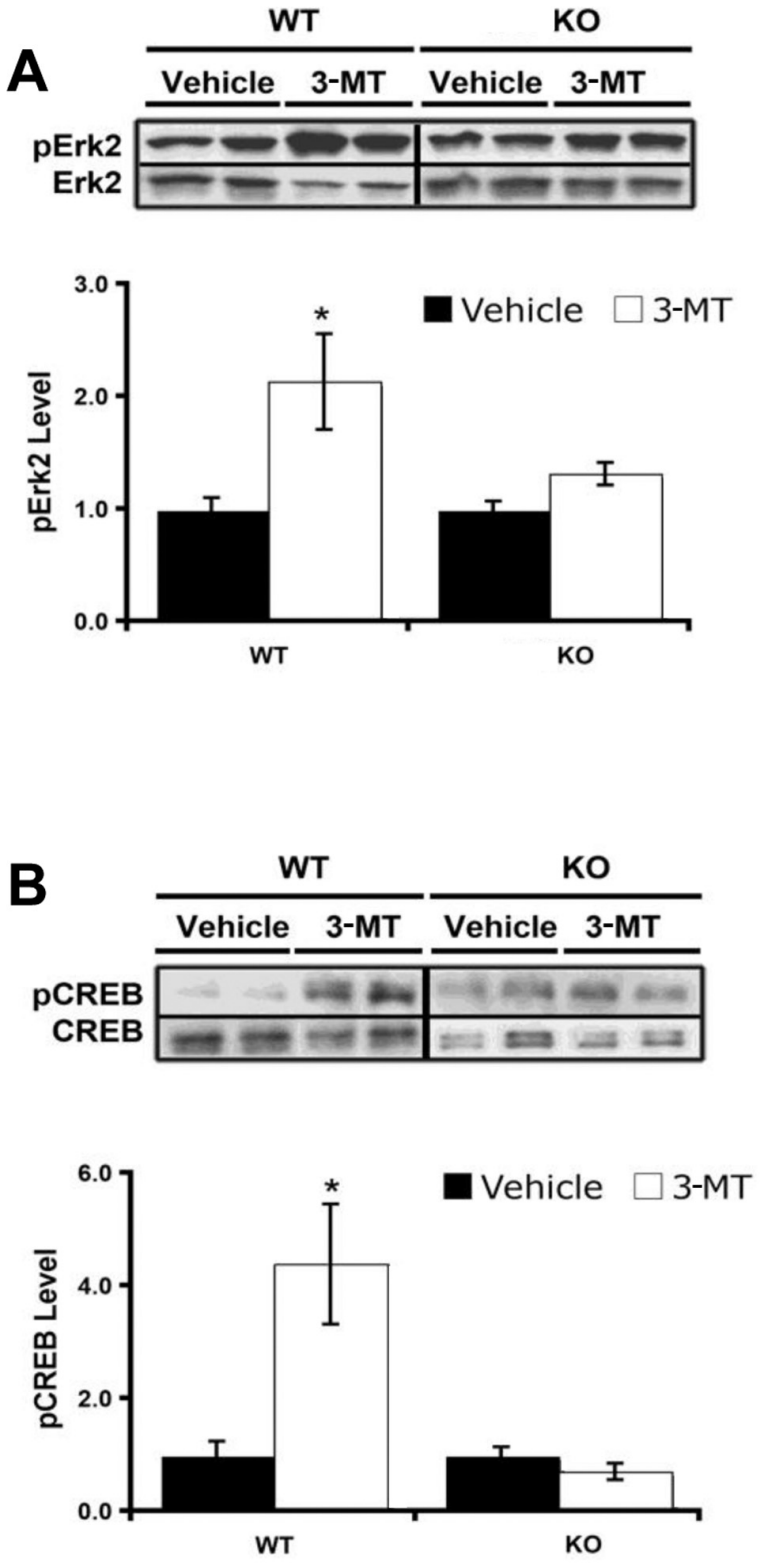
Striatal signaling effects of 3-MT are reduced in TAAR1-KO mice. Effect of 3-MT infusion (18 µg, 30 min after treatment) on Erk2 (A) and CREB (B) phosphorylation in WT and TAAR1-KO mice. 3-MT induced significant phosphorylation of both Erk2 and CREB in WT mice, but not in TAAR1-KO mice (* p<0.05; Student's t-test n = 10 per group).

To directly test if TAAR1 is involved in the action of 3-MT we analyzed behavioral and biochemical responses to 3-MT in mice lacking TAAR1 [32]. As presented in the Figure 3, i.c.v. infusion of 3-MT (9,18 and 36 µg) induced a dose-dependent activation of wild type mice with a complex set of often competing abnormal behaviors as reflected by changes in automated measures of movement time, total distance traveled and vertical activity (Figure 3, Supplemental Videos S1 and S2). However, in TAAR1-KO mice these effects of 3-MT were reduced (Figure 3C,D,E) with the lowest dose (9 µg) being completely inactive in the mutants (Figure 3A,B). However at highest dose tested, 3-MT still caused significant behavioral activation in TAAR1 mutants and similar, albeit somewhat reduced in mutants, pattern of abnormal movements was observed in both genotypes after 36 µg of 3-MT (Figure 3; visual observations). Furthermore, analysis of Erk2 and CREB phosphorylation in the striatum provoked by an intermediate dose of 3-MT (18 µg) that causes significant behavioral activation in wild type mice (Figure 3C,D,E), revealed that these signaling events were significantly reduced in TAAR1 mutants (Figure 4). Thus, it is likely that part of the behavioral activation and a significant portion of striatal signaling events triggered by 3-MT are dependent on TAAR1. However, the fact that only a part of 3-MT effects was affected by TAAR1 deficiency strongly suggests that other non TAAR1-mediated mechanisms are also involved in the action of 3-MT.

## Discussion

In this study we demonstrate that 3-MT, the major product of extracellular dopamine metabolism mediated by COMT, can directly affect behavior and induce intracellular signaling events partially via activation of TAAR1. Importantly, this action of 3-MT does not directly involve dopaminergic transmission and can be observed in mice lacking DA. At the same time, effects of 3-MT are partially reduced in mice lacking TAAR1 indicating a role of TAAR1 in physiological actions of 3-MT. Taken together, these observations suggest that rather than being just an inactive metabolite of extracellular DA, 3-MT is a neuromodulator that at high concentrations can exert physiological actions partially via activating TAAR1.

### 3-MT as a neuromodulator

3-MT is a well known extracellular metabolite of 3-hydroxytyramine/dopamine. Historically, dopamine was considered as a biologically inactive precursor of norepinephrine and only the identification of large concentrations of dopamine in certain brain regions and the direct demonstration of its physiological functions particularly in movement control [33], [34] firmly established dopamine as the classical monoaminergic neurotransmitter/neuromodulator. A major source of 3-MT is the released dopamine in the extracellular space that is being metabolized via o-methylation by COMT to yield 3-MT [1]. A predominant mechanism of inactivation of 3-MT is its MAO mediated metabolism to homovanillic acid (HVA), that in turn is cleared from the brain by specific transporters [35], [36]. Since extracellular concentrations of DA are generally found in a low nanomolar range, concentrations of 3-MT at steady state in the brain are also quite low [7], [37]. The fact that 3-MT originates from released dopamine has led to consideration of 3-MT levels as a reflection of DA release. In fact, numerous studies have been performed on the analysis of DA release in various experimental paradigms by assessing accumulation of 3-MT tissue levels in brain tissue, often after blockade of MAO by pargyline [7], [38]. However, while later in vivo microdialysis studies have generally confirmed the association between extracellular levels of DA and 3-MT [37], the static tissue measures of 3-MT accumulation have proven to be quite inaccurate measures of dynamics of DA release [39], [40].

In our unbiased screen for potential dopamine-independent actions of putative TAAR1 ligands on the movement control in DDD mice, we failed to detect significant effects of trace amines and major metabolites of monoamines ([12]; Table 1). The only exception was 3-MT, which caused a complex set of abnormal movements in DDD mice. It should be noted that while there is a common belief that 3-MT is just an inactive metabolite of dopamine, several groups have reported specific behavioral effects of 3-MT in experimental animals, ranging from 3-MT-induced tremor [41], stereotypies [42], [43], [44], hyperactivity [43], [44] and even hypoactivity [45]. The present study unequivocally shows behavioral and intracellular signaling effects of this endogenous compound and identifies TAAR1 as one of the mediators of 3-MT actions *in vivo*. It should be noted, however, that under normal conditions the basal concentrations of 3-MT in the extracellular space appear relatively low (below 10 nM) [10] to significantly affect TAAR1 since the affinity of 3-MT at TAAR1 under different *in vitro* conditions was found to be in the range of 350-2,810 nM ([16], [20], [21], [22], present study). However, a similar situation exists for the dopamine system where steady state 7–10 nM concentrations of dopamine are detected by in vivo microdialysis [10] but, for example, the affinity of DA at D1 DA receptors is routinely reported to be in the micromolar range [46]. Direct microdialysis measurements of the extracellular concentrations of 3-MT following i.c.v. administration have shown that infusion of a behaviorally active dose of 3-MT (9 µg, i.c.v.) results in accumulation of about 100 nM concentration of 3-MT in the striatal dialysates (Figure S2). Given the fact that the *in vitro* recovery of 3-MT across the dialysis membrane at present conditions is about 20% (data not shown), we estimate that behaviorally active extracellular 3-MT concentrations should be above 500 nM which is consistent with affinity values of 3-MT at TAAR1 in cellular assays ([16], [20], [21], [22], Figure 2). Since the spatial and temporal dynamics of 3-MT concentrations following the release of DA in the synaptic cleft are unknown [47], it is unclear if 3-MT under normal conditions achieves the concentrations required to activate TAAR1. However, in pathological situations or with pharmacological treatments causing massive DA release or a deficiency in MAO, accumulated 3-MT could possibly reach the concentrations that would activate TAAR1. Similarly, decreases in 3-MT concentrations due to central COMT inhibition could significantly diminish the possibility of TAAR1-mediated effects of 3-MT.

Another important question relates to the fact that the effects of 3-MT were only partially blocked in TAAR1-KO mice. This observation indicates that TAAR1 is not the exclusive receptor involved in the actions of 3-MT and a complete explanation of the behavioral effects and, to a lesser degree, striatal signaling responses requires the additional actions on receptors other than TAAR1. While there is some indication that at high micromolar concentrations 3-MT may bind to dopamine and α2-adrenergic receptors [48], [49], it is more likely that other, currently unrecognized receptors could play important role in 3-MT effects. It should be noted, that the TAAR receptor gene family in humans is represented by 9 members and in mice by 16 members [17] and it is not inconceivable that the full physiological output of a monoaminergic neuromodulator/neurotransmitter requires the activation of multiple receptors of the same family. For example, activation of both D1 and D2 dopamine receptors is necessary to induce movement in conditions of dopamine deficiency [12].

### Novel roles of COMT and MAO

3-MT concentrations in the brain are tightly controlled by the activity of COMT and MAO (with major contribution of MAO-A) [1], [35], [38], [50], [51]. As such, inhibitors of COMT lead to dramatic decreases in 3-MT levels while blockade of MAO induces remarkable elevations in 3-MT levels [35], [38], [50], [52]. Intriguingly, both COMT and MAO inhibitors cause significant modulating effects on the clinical manifestations of Parkinson's disease [6], [53]. Polymorphisms in *MAOA* gene have been associated with aggression, affective disorders, alcoholism and attention deficit hyperactivity disorder (ADHD) [54], [55], [56], [57]. Variations in *COMT* gene affecting enzyme activity has been associated with schizophrenia, ADHD, pain sensitivity and several other pathological conditions [6], [55], [56], [58], [59] but the role of this enzyme has been exclusively related to modulation of metabolism of classical catecholamines, such as dopamine and norepinephrine. The realization of the fact that 3-MT has its own neuromodulatory properties suggests that alterations in COMT activity, which serves as the rate limiting enzyme for this putative neuromodulator, could affect brain functions also by altering extracellular 3-MT concentrations. Understanding the role of 3-MT mediated effects of COMT and MAO in various pathological conditions represents an exciting topic for future research.

### Potential role of 3-MT in Parkinson's disease and schizophrenia

Both COMT and MAO inhibitors have found clinical utility in Parkinson's disease [6], [53]. It is tempting to speculate that the behavioral effects caused by 3-MT may have particular relevance to the pathogenesis or responses to treatments in this disorder. It has been suggested previously that abnormal 3-MT levels may contribute to some side-effects of L-DOPA treatment [43]–[45]. Particularly intriguing is the observation that 3-MT concentrations are more markedly increased in the putamen of patients that develop L-DOPA-induced dyskinesia [60]. The low nanomolar concentrations of 3-MT, closely following the dynamics of extracellular DA levels, should be elevated by each administration of L-DOPA [61]. It is unknown what concentrations of 3-MT in the brain are produced by the chronic multi-year treatment of L-DOPA that is necessary to cause dyskinesias in PD patients, but a large increase in urine 3-MT levels have been reported in patients treated chronically with L-DOPA [62], [63]. Thus, a potential contribution of elevated 3-MT levels to at least some specific manifestations of L-DOPA-induced dyskinesias and the role of TAAR1 in these processes deserve further detailed investigation.

A dopaminergic theory of schizophrenia suggests an enhanced dopaminergic transmission as a leading cause of the disorder. An enhanced dopamine release should produce elevation in brain 3-MT concentrations and variations in COMT activity found in this disorder [59] could significantly affect these levels. Thus, it might be important to explore whether TAAR1-dependent neuromodulation caused by 3-MT contributes to pathological manifestations of schizophrenia [64].

### Conclusions

Taken together, these observations indicate an important neuromodulatory role for the major extracellular dopamine metabolite, 3-MT. The data suggest the broadening of the list of tyrosine metabolites – dopamine, norepinephrine, epinephrine, tyramine, octopamine, β-PEA, and now 3-MT– that can exert significant neuromodulatory/neurotransmitter actions in various organisms. The identification of 3-MT as a neuromodulator supports the concept that multiple products of a single synthetic pathway and also their degradation products can serve as signals to affect specific neuronal systems or provide mechanisms for the fine-tuning of physiological functions. Finally, given low (trace) concentrations of 3-MT at steady state in the brain, its phenylethylamine structure and activity at TAAR1, this biogenic amine could be classified as a novel member of the family of endogenous trace amines.

## Supporting Information

Figure S1 Automated measures of dynamics of abnormal movements induced by 3-MT in wild type mice.
(0.04 MB PDF)

Figure S2 Determination of striatal extracellular levels of 3-MT after i.c.v. infusion of 3-MT (9 µg) into the lateral ventricle.
(0.02 MB PDF)

Video S1 Examples of abnormal motor behaviors induced by 3-MT (36 µg, i.c.v.) in wild type mice (40 minutes after administration).
(6.70 MB MPG)

Video S2 Examples of abnormal motor behaviors induced by 3-MT (36 µg, i.c.v.) in wild type mice (40 minutes after administration).
(8.39 MB WMV)

## References

[1] Molinoff PB, Axelrod J (1971). Biochemistry of catecholamines.. Annu Rev Biochem.

[2] Zhou QY, Palmiter RD (1995). Dopamine-deficient mice are severely hypoactive, adipsic, and aphagic.. Cell.

[3] Carlsson A (1972). Biochemical and pharmacological aspects of Parkinsonism.. Acta Neurol Scand.

[4] Fon EA, Pothos EN, Sun BC, Killeen N, Sulzer D (1997). Vesicular transport regulates monoamine storage and release but is not essential for amphetamine action.. Neuron.

[5] Wang YM, Gainetdinov RR, Fumagalli F, Xu F, Jones SR (1997). Knockout of the vesicular monoamine transporter 2 gene results in neonatal death and supersensitivity to cocaine and amphetamine.. Neuron.

[6] Mannisto PT, Kaakkola S (1999). Catechol-O-methyltransferase (COMT): biochemistry, molecular biology, pharmacology, and clinical efficacy of the new selective COMT inhibitors.. Pharmacol Rev.

[7] Westerink BH, Spaan SJ (1982). On the significance of endogenous 3-methoxytyramine for the effects of centrally acting drugs on dopamine release in the rat brain.. J Neurochem.

[8] Amara SG, Sonders MS (1998). Neurotransmitter transporters as molecular targets for addictive drugs.. Drug Alcohol Depend.

[9] Giros B, Jaber M, Jones SR, Wightman RM, Caron MG (1996). Hyperlocomotion and indifference to cocaine and amphetamine in mice lacking the dopamine transporter.. Nature.

[10] Jones SR, Gainetdinov RR, Jaber M, Giros B, Wightman RM (1998). Profound neuronal plasticity in response to inactivation of the dopamine transporter.. Proc Natl Acad Sci U S A.

[11] Gainetdinov RR, Caron MG (2003). Monoamine transporters: from genes to behavior.. Annu Rev Pharmacol Toxicol.

[12] Sotnikova TD, Beaulieu JM, Barak LS, Wetsel WC, Caron MG (2005). Dopamine-independent locomotor actions of amphetamines in a novel acute mouse model of Parkinson disease.. PLoS Biol.

[13] Sotnikova TD, Caron MG, Gainetdinov RR (2006). DDD mice, a novel acute mouse model of Parkinson's disease.. Neurology.

[14] Sotnikova TD, Zorina OI, Ghisi V, Caron MG, Gainetdinov RR (2008). Trace amine associated receptor 1 and movement control.. Parkinsonism Relat Disord.

[15] Borowsky B, Adham N, Jones KA, Raddatz R, Artymyshyn R (2001). Trace amines: identification of a family of mammalian G protein-coupled receptors.. Proc Natl Acad Sci U S A.

[16] Bunzow JR, Sonders MS, Arttamangkul S, Harrison LM, Zhang G (2001). Amphetamine, 3,4-methylenedioxymethamphetamine, lysergic acid diethylamide, and metabolites of the catecholamine neurotransmitters are agonists of a rat trace amine receptor.. Mol Pharmacol.

[17] Lindemann L, Hoener MC (2005). A renaissance in trace amines inspired by a novel GPCR family.. Trends Pharmacol Sci.

[18] Grandy DK (2007). Trace amine-associated receptor 1-Family archetype or iconoclast?. Pharmacol Ther.

[19] Sotnikova TD, Caron MG, Gainetdinov RR (2009). Trace amine-associated receptors as emerging therapeutic targets.. Mol Pharmacol.

[20] Wainscott DB, Little SP, Yin T, Tu Y, Rocco VP (2007). Pharmacologic characterization of the cloned human trace amine-associated receptor1 (TAAR1) and evidence for species differences with the rat TAAR1.. J Pharmacol Exp Ther.

[21] Barak LS, Salahpour A, Zhang X, Masri B, Sotnikova TD (2008). Pharmacological characterization of membrane-expressed human trace amine-associated receptor 1 (TAAR1) by a bioluminescence resonance energy transfer cAMP biosensor.. Mol Pharmacol.

[22] Hu LA, Zhou T, Ahn J, Wang S, Zhou J (2009). Human and mouse trace amine-associated receptor 1 have distinct pharmacology towards endogenous monoamines and imidazoline receptor ligands.. Biochem J.

[23] Osborne RH (1996). Insect neurotransmission: neurotransmitters and their receptors.. Pharmacol Ther.

[24] Roeder T (2005). Tyramine and octopamine: ruling behavior and metabolism.. Annu Rev Entomol.

[25] Beaulieu JM, Sotnikova TD, Yao WD, Kockeritz L, Woodgett JR (2004). Lithium antagonizes dopamine-dependent behaviors mediated by an AKT/glycogen synthase kinase 3 signaling cascade.. Proc Natl Acad Sci U S A.

[26] Gainetdinov RR, Wetsel WC, Jones SR, Levin ED, Jaber M (1999). Role of serotonin in the paradoxical calming effect of psychostimulants on hyperactivity.. Science.

[27] Salomon Y, Londos C, Rodbell M (1974). A highly sensitive adenylate cyclase assay.. Anal Biochem.

[28] Beaulieu JM, Marion S, Rodriguiz RM, Medvedev IO, Sotnikova TD (2008). A beta-arrestin 2 signaling complex mediates lithium action on behavior.. Cell.

[29] Beaulieu JM, Sotnikova TD, Gainetdinov RR, Caron MG (2006). Paradoxical striatal cellular signaling responses to psychostimulants in hyperactive mice.. J Biol Chem.

[30] Girault JA, Valjent E, Caboche J, Herve D (2007). ERK2: a logical AND gate critical for drug-induced plasticity?. Curr Opin Pharmacol.

[31] Santini E, Alcacer C, Cacciatore S, Heiman M, Herve D (2009). L-DOPA activates ERK signaling and phosphorylates histone H3 in the striatonigral medium spiny neurons of hemiparkinsonian mice.. J Neurochem.

[32] Wolinsky TD, Swanson CJ, Smith KE, Zhong H, Borowsky B (2006). The Trace Amine 1 receptor knockout mouse: an animal model with relevance to schizophrenia.. Genes Brain Behav.

[33] Carlsson A, Lindqvist M, Magnusson T, Waldeck B (1958). On the presence of 3-hydroxytyramine in brain.. Science.

[34] Birkmayer W, Hornykiewicz O (1961). [The L-3,4-dioxyphenylalanine (DOPA)-effect in Parkinson-akinesia.].. Wien Klin Wochenschr.

[35] Westerink BH, Spaan SJ (1982). Estimation of the turnover of 3-methoxytyramine in the rat striatum by HPLC with electrochemical detection: implications for the sequence in the cerebral metabolism of dopamine.. J Neurochem.

[36] Mori S, Takanaga H, Ohtsuki S, Deguchi T, Kang YS (2003). Rat organic anion transporter 3 (rOAT3) is responsible for brain-to-blood efflux of homovanillic acid at the abluminal membrane of brain capillary endothelial cells.. J Cereb Blood Flow Metab.

[37] Brown EE, Damsma G, Cumming P, Fibiger HC (1991). Interstitial 3-methoxytyramine reflects striatal dopamine release: an in vivo microdialysis study.. J Neurochem.

[38] Kehr W (1981). 3-Methoxytyramine and normetanephrine as indicators of dopamine and noradrenaline release in mouse brain in vivo.. J Neural Transm.

[39] Vulto AG, Westenberg HG, Meijer LB, Versteeg DH (1986). The dopamine metabolite 3-methoxytyramine is not a suitable indicator of dopamine release in the rat brain.. J Neurochem.

[40] Elverfors A, Pileblad E, Lagerkvist S, Bergquist F, Jonason J (1997). 3-Methoxytyramine formation following monoamine oxidase inhibition is a poor index of dendritic dopamine release in the substantia nigra.. J Neurochem.

[41] Baker WW, Zivanovic D, Malseed RT (1976). Tremorogenic effects of intracaudate d-amphetamine and their suppression by dopamine.. Arch Int Pharmacodyn Ther.

[42] Jonas W, Scheel-Kruger J (1969). Amphetamine induced stereotyped behaviour correlated with the accumulation of O-methylated dopamine.. Arch Int Pharmacodyn Ther.

[43] Nakazato T (2002). The medial prefrontal cortex mediates 3-methoxytyramine-induced behavioural changes in rat.. Eur J Pharmacol.

[44] Nakazato T, Akiyama A (2002). Behavioral activity and stereotypy in rats induced by L-DOPA metabolites: a possible role in the adverse effects of chronic L-DOPA treatment of Parkinson's disease.. Brain Res.

[45] Charlton CG, Crowell B (2000). Effects of dopamine metabolites on locomotor activities and on the binding of dopamine: relevance to the side effects of L-dopa.. Life Sci.

[46] Missale C, Nash SR, Robinson SW, Jaber M, Caron MG (1998). Dopamine receptors: from structure to function.. Physiol Rev.

[47] Garris PA, Wightman RM (1995). Distinct pharmacological regulation of evoked dopamine efflux in the amygdala and striatum of the rat in vivo.. Synapse.

[48] Antkiewicz-Michaluk L, Ossowska K, Romanska I, Michaluk J, Vetulani J (2008). 3-Methoxytyramine, an extraneuronal dopamine metabolite plays a physiological role in the brain as an inhibitory regulator of catecholaminergic activity.. Eur J Pharmacol.

[49] Alachkar A, Brotchie JM, Jones OT (2010). Binding of dopamine and 3-methoxytyramine as l-DOPA metabolites to human alpha(2)-adrenergic and dopaminergic receptors.. Neurosci Res.

[50] Kato T, Dong B, Ishii K, Kinemuchi H (1986). Brain dialysis: in vivo metabolism of dopamine and serotonin by monoamine oxidase A but not B in the striatum of unrestrained rats.. J Neurochem.

[51] Lenders JW, Eisenhofer G, Abeling NG, Berger W, Murphy DL (1996). Specific genetic deficiencies of the A and B isoenzymes of monoamine oxidase are characterized by distinct neurochemical and clinical phenotypes.. J Clin Invest.

[52] Mannisto PT, Tuomainen P, Tuominen RK (1992). Different in vivo properties of three new inhibitors of catechol O-methyltransferase in the rat.. Br J Pharmacol.

[53] Youdim MB, Edmondson D, Tipton KF (2006). The therapeutic potential of monoamine oxidase inhibitors.. Nat Rev Neurosci.

[54] Shih JC, Thompson RF (1999). Monoamine oxidase in neuropsychiatry and behavior.. Am J Hum Genet.

[55] D'Souza UM, Craig IW (2008). Functional genetic polymorphisms in serotonin and dopamine gene systems and their significance in behavioural disorders.. Prog Brain Res.

[56] Haavik J, Blau N, Thony B (2008). Mutations in human monoamine-related neurotransmitter pathway genes.. Hum Mutat.

[57] Gizer IR, Ficks C, Waldman ID (2009). Candidate gene studies of ADHD: a meta-analytic review.. Hum Genet.

[58] Diatchenko L, Nackley AG, Slade GD, Bhalang K, Belfer I (2006). Catechol-O-methyltransferase gene polymorphisms are associated with multiple pain-evoking stimuli.. Pain.

[59] Apud JA, Weinberger DR (2007). Treatment of cognitive deficits associated with schizophrenia: potential role of catechol-O-methyltransferase inhibitors.. CNS Drugs.

[60] Rajput AH, Fenton ME, Di Paolo T, Sitte H, Pifl C (2004). Human brain dopamine metabolism in levodopa-induced dyskinesia and wearing-off.. Parkinsonism Relat Disord.

[61] Napolitano A, Zurcher G, Da Prada M (1995). Effects of tolcapone, a novel catechol-O-methyltransferase inhibitor, on striatal metabolism of L-dopa and dopamine in rats.. Eur J Pharmacol.

[62] Siirtola T, Sonninen V, Rinne UK (1975). Urinary excretion of monoamines and their metabolites in patients with Parkinson's disease. Response to long-term treatment with levodopa alone or in combination with a dopa decarboxylase inhibitor and clinical correlations.. Clin Neurol Neurosurg.

[63] Muskiet FA, Thomasson CG, Gerding AM, Fremouw-Ottevangers DC, Nagel GT (1979). Determination of catecholamines and their 3-O-methylated metabolites in urine by mass fragmentography with use of deuterated internal standards.. Clin Chem.

[64] Dill RE, Campbell KM (1973). 3 methoxytyramine: a possible endogenous toxin of psychosis?. Res Commun Chem Pathol Pharmacol.

