# Correction: The Dopamine Metabolite 3-Methoxytyramine Is a Neuromodulator

**DOI:** 10.1371/annotation/a2019934-b1cc-4781-80cb-9e09fc7ff6dc

**Published:** 2010-10-28

**Authors:** Tatyana D. Sotnikova, Jean-Martin Beaulieu, Stefano Espinoza, Bernard Masri, Xiaodong Zhang, Ali Salahpour, Larry S. Barak, Marc G. Caron, Raul R. Gainetdinov

There was an error in affiliation 4. The correct address is: 4 INSERM U 858 and Université Paul Sabatier, Toulouse, France. Also, the following information was omitted from the funding information: "Bernard Masri is recipient of a European Marie Curie Outgoing International Fellowship (FP6-2005-Mobility 6)."

