# Supplementary material for: The Dopamine Metabolite 3-Methoxytyramine Is a Neuromodulator
Source: PLoS One. 2010 Oct 18;5(10):e13452. doi: 10.1371/journal.pone.0013452 (PMC2956650; doi:10.1371/journal.pone.0013452)
Supplement: Figure S1 — Automated measures of dynamics of abnormal movements induced by 3-MT in wild type mice. (0.04 MB PDF) [file pone.0013452.s001.pdf]

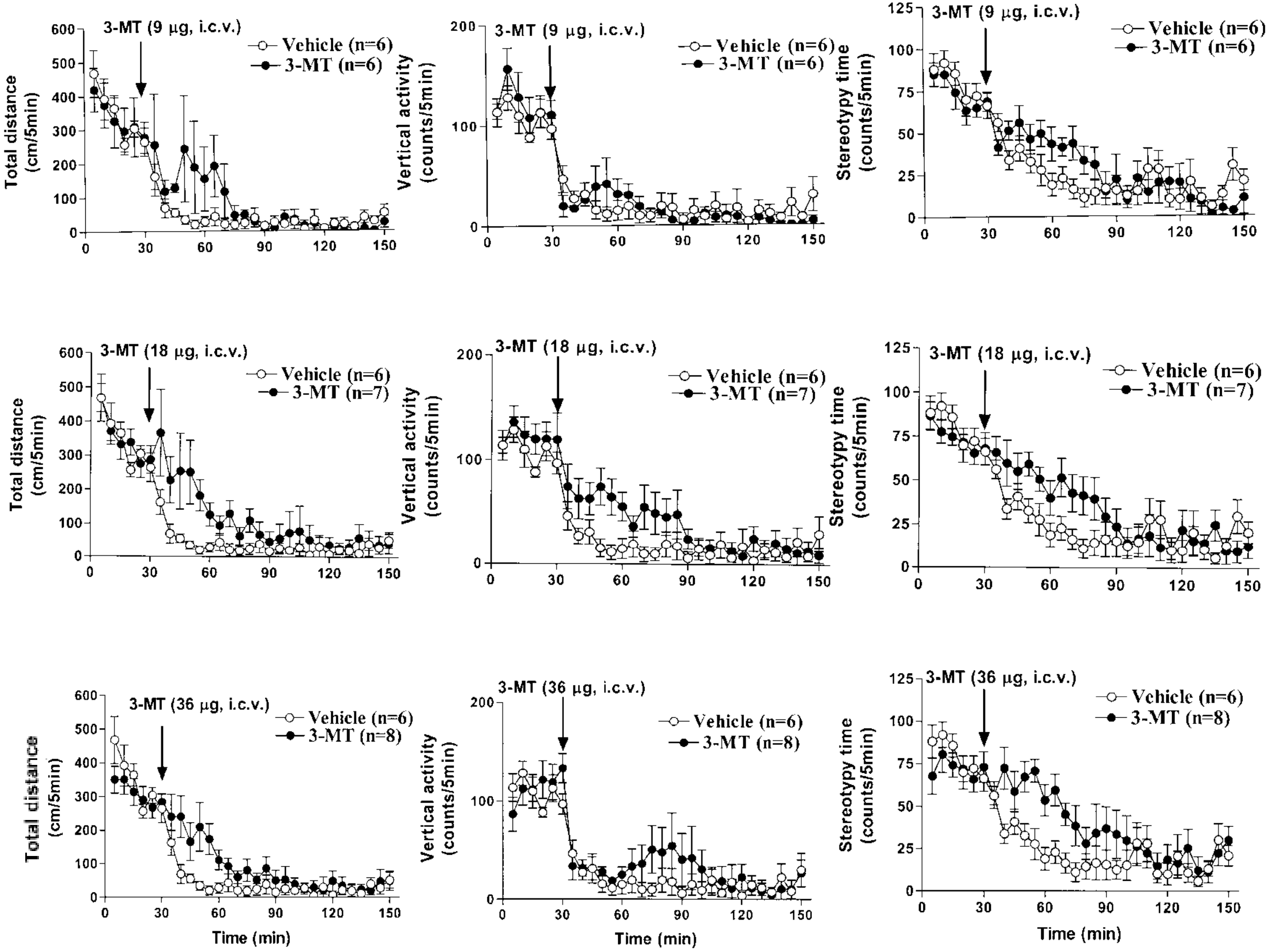

Supplemental Figure S1 - Automated measures of dynamics of abnormal movements induced by 3-MT in wild type mice.
3-MT was infused i.c.v. at doses 9, 18 and 36 µg to habituated male C57Bl6 mice and various parameters of motor activity were detected by computerized locomotor activity monitor. Dynamics of abnormal movements caused by 3-MT are presented as changes in total distance traveled, vertical activity and stereotypy time. Please see a description in the text and Supplemental Videos 1 and 2 for detailed description of abnormal involuntary movements triggered by 3-MT. Experiments were performed in 6-8 mice per group.
