# Supplementary material for: The Dopamine Metabolite 3-Methoxytyramine Is a Neuromodulator
Source: PLoS One. 2010 Oct 18;5(10):e13452. doi: 10.1371/journal.pone.0013452 (PMC2956650; doi:10.1371/journal.pone.0013452)
Supplement: Figure S2 — Determination of striatal extracellular levels of 3-MT after i.c.v. infusion of 3-MT (9 µg) into the lateral ventricle. (0.02 MB PDF) [file pone.0013452.s002.pdf]

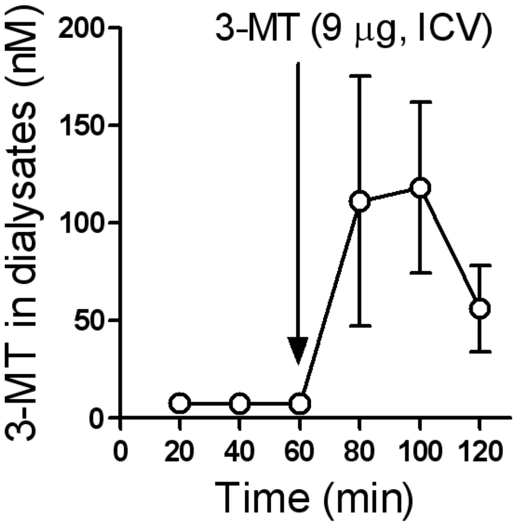

Supplemental Figure S2 - Determination of striatal extracellular levels of 3-MT after i.c.v. infusion of 3-MT (9 µg) into the lateral ventricle. *In vivo* microdialysis experiments were performed in freely moving C57Bl6 mice as described [10,12]. Simultaneously with implantation of cannulas for i.c.v. infusion, microdialysis probes were implanted to the right striatum of anaesthetized mice and 48 hours after operation, 3-MT was infused into the right lateral ventricle. Microdialysis samples were collected for at least 60 min before and 60 min after infusion and analyzed by HPLC for dialysate 3-MT levels as described [10,12]. Experiments were performed in 6 mice.
